# A Case

**Published:** 1864-11

**Authors:** Gam’l Jackson


### A CASE.

BY GAM'L JACKSON.

A gentleman called at my office a year and a half ago to have his teeth examined, particularly the right superior first bicuspid. The nerve of the tooth had been destroyed, and the fangs and crown had been filled with gold one year before. The plug was nearly two-thirds as large as the entire crown; the buccal and lingual walls, and half of the distal only remaining. In making this plug the Dentist had succeeded in splitting the tooth, dividing the crown fairly between the cusps, the split diverging beneath the gum obliquely across the buccal fang, and terminating at about one-third its length on the buccal side. The plug was loose and easily removed. A cut of rubber tubing was placed on the tooth, and in two days the parts which had become permanently separated, were brought perfectly together. My patient was here called away on business, and when he returned, three weeks afterwards, the case was complicated with abscess. One week's treatment resulted favorably, but the smaller piece of the tooth was left quite loose. The operation was completed by putting an ordinary shaped bolt made from No. 18, hard gold wire through the tooth, drilling from about the middle of the buccal surface. The bolt was put through from the lingual side, so that the nut could be readily tightened to hold the parts together. The cavity of the divided fang was filled with cotton, moistened with creosote, and the crown with Wood's Filling. The end of the bolt was burnished to a head over the nut, and the case was dismissed. I examined this tooth a few days since and found it substantially as when I left it eighteen months ago, and my patient reports his perfect satisfaction with it.

